# Annotations

**Published:** 1906-09-08

**Authors:** 


### ANNOTATIONS.

### Medical "Warnings."

THE analytical chemist and the medical practitioner, alone or in combination, certainly keep a keen outlook on the possible avenues from which danger to health may approach either the individual citizen or the community at large. Their care in this direction is not always appreciated, and it has occasionally exhibited itself in the shape of sensational statements which receive successful contradiction from the experience of every-day life. Such excess of zeal, or lack of perspective, is much to be regretted, for not only does it give occasion for the enemy to blaspheme, but it weakens the whole body of prophylactic doctrine upon which the preservation of the public health may be said largely to rest. To issue in startling terms a warning against some public practice or habit without an adequate scientific support to justify the implied conclusion is doubtless a temptation to those ambitious of a cheap notoriety. Nevertheless, such a proceeding ought to be condemned as not much less than a social crime. Alertness in observation and interpretation are, of course, to be welcomed and commended, but, on the whole, we believe the cause of the public health would be better served were anything of the nature of a medical pronouncement against dietetic or other practices as injurious to health reserved for some authoritative body, such, for example, as the Local Government Board. It is impossible to deny that the daily press, acting, at least sometimes, on professed medical inspiration, does much harm by the alarming reports some of its representatives delight to publish. Poisons in hat linings, in stockings, in india-rubber stoppers, and a thousand other dangers form the text for a brief sensation which endures until the next "cause" of appendicitis, or whatever it may be, is discovered. All this, though much of it has no other claim to attention than the "belief" or "view" of some talkative nobody, is meat and drink to those who cultivate conversation in health as a constant entertainment; but in the judgment of sensible persons it is apt to weaken the serious significance of medical opinion. It would be well if the emphatic condemnation of the profession could be recorded against those of its members who promote the publication of unwarranted, or even unproved, medical "warnings."

### Quatro-centenary of the University of Aberdeen.

THE quatro-centenary of the foundation of the University of Aberdeen is to be celebrated in a series of academic and civic demonstrations, which are to extend from the 25th to the 28th of the present month. By a happy chance this considerable event in the history of the University coincides with the completion of the new buildings at Marischall College—a scheme which has occupied the attention of the authorities for several years. These buildings not only provide the accommodation necessary for the demands of modern medical and scientific teaching, but they possess the additional attraction of great architectural nobility and grace. Not unnaturally their inauguration will be one of the most conspicuous features of the forthcoming celebrations, and this all the more that His Majesty the King has graciously signified his intention of performing the inauguration ceremony. In addition to the interesting and central event the programme for the four days over which the celebrations are to extend includes formal receptions at the University and at the Royal Infirmary, and also an academic ceremony for the conferring of honorary degrees on a number of the official delegates. Town and gown are to vie with one another in hospitality to their visitors, and the students are to have abundant opportunities to assert themselves in the shape of a ball, a symposium, and a torch-light procession. Altogether the celebrations are to assume memorable proportions—worthy of the traditions of a great university and of the distinction, enterprise, and loyalty of her sons. In offering our congratulations, we beg also to add the expression of our sincere good wishes for the future.

### Sea-Bathing and Ear Complaints.

THIS is the bathing season and the season of acute inflammatory middle ear disease. Every worker in an ear clinique knows how frequently the sufferer from middle ear suppuration refers his disease to sea bathing. Not a few cases of extension of already existing disease to the mastoid cells are caused by a cold douche of sea-water. The cases most prone to serious ear symptoms after bathing are, as might be expected, those already suffering from chronic ear discharge. If, on the other hand, there is no chronic suppuration, and if the drum-membranes are intact, sea-bathing is almost quite harmless so far as the ear is concerned. But where the membranes have been perforated, whereby an entrance is provided for the sea-water into the delicate middle ear, the risk of acute middle ear suppuration is quite considerable. We may suppose that the cold affusion lowers the resistance of the tissues, so that an invasion of the pus-producing organisms is rendered possible, but all around our coasts pyogenic organisms exist in sufficient numbers in sea-water to set up direct infection. Prophylaxis is simple. Bathers who already suffer from otorrhœa, and those who have been subjects of the disease in the past, should entirely abstain from bathing, or, if they do bathe, should protect their ears by inserting plugs of non-absorbing cotton-wool into the external auditory meatus. For this purpose, perhaps the most serviceable and efficient plug is one made of "plasticine," or some similar clay, in which is worked up some cotton-wool. The instrument makers sell these plugs under the name of "sound deadeners."

